# Surface Modification of Carbon Fiber for Enhancing the Mechanical Strength of Composites

**DOI:** 10.3390/polym14193999

**Published:** 2022-09-24

**Authors:** Ryoma Tokonami, Katsuhito Aoki, Teruya Goto, Tatsuhiro Takahashi

**Affiliations:** Department of Organic Materials Science, Graduated School of Organic Materials Science, Yamagata University, 4-3-16 Jonan, Yonezawa 992-8510, Yamagata, Japan

**Keywords:** composite, carbon fiber, interface, carbon nanotubes, reactive polymer, layer-by-layer

## Abstract

The surface of carbon fibers (CFs) is often modified by multi-walled carbon nanotubes (MWCNTs), and the effect of the interface on the mechanical properties has been reported mostly for epoxy matrices. We achieved effective surface modification of CFs by a simple two-step process to graft a large amount of MWCNTs using a highly reactive polymer to enhance the bonding between CFs and MWCNTs. The first step was the reactive mono-molecular coating of a reactive polymer (poly-2-isopropenyl-2-oxazoline; Pipozo) that has high reactivity with COOH from CFs and MWCNTs. The high reactivity between the oxazoline group and COOH or phenol OH was confirmed for low-molecular-weight reactions. The second step was the coating of MWCNTs from a dispersion in a solvent. This simple process resulted in a substantial amount of MWCNTs strongly bonded to CF, even after washing. The MWCNTs grafted onto CFs remained even after melt-mixing. The effect on the interface, i.e., physical anchoring, led to an improvement of the mechanical properties. The novelty of the present study is that Pipozo acted as a molecular bonding layer between CFs and MWCNTs as a physical anchoring structure formed by a simple process, and the interface caused a 20% improvement in the tensile strength and modulus. This concept of a composite having a physical anchoring structure of MWCNTs on CFs has potential applications for lightweight thermoplastics, such as in the automotive industry.

## 1. Introduction

Carbon fiber-reinforced plastics (CFRP) are attractive with respect to their strong mechanical properties, lightweight, long continuous, or short carbon fibers (CFs), and are fabricated based on either thermosetting or a thermoplastic matrix. Research on composites including multi-walled carbon nanotubes (MWCNTs) in a matrix together with CF has been conducted with the aim of a synergistic effect of MWCNTs and CFs to enhance the mechanical strength [1,2,3,4]. On the other hand, to improve the mechanical properties of CFRP, many approaches focused on the surface treatment of CFs have been reported, the treatments of which can be categorized as wet, dry, nano, oxidation, and non-oxidation, as described in a recent review article [5]. Related with the surface technology, surface analysis methods, surface control, and surface modification were described in another review article [6]. There have been reports on the use of amines to achieve a chemical reaction between CFs and a matrix polymer [7,8,9]. However, it is considered that there would not be very effective chemical bonding between amines and carboxylic acid because of the fundamental characteristics of the reversible reaction at equilibrium, even when using a catalyst.

As a typical method for the surface modification of CFs with nanomaterials, there is the surface modification of CFs using MWCNTs. To coat carbon nanotubes (CNTs) onto CF surfaces, various methods such as chemical vapor deposition (CVD) [10,11,12,13], electrophoretic deposition (EPD) [14], and chemical functionalization [15,16] were reported in the review article by Zakaria et al. [17]. Surfactants with either high or low molecular weight have been utilized for the chemical functionalization method [17]. Another review article reported CVD, a spray-coating method, and dip coating [18,19] methods using MWCNTs [20]. Pezegic et al. grew MWCNTs on long continuous CF surfaces by the CVD method and to prepare a composite using these CFs with infusion molding for the evaluation of electric and thermal conductivities [10]. Boroujeni et al. grew MWCNTs by the CVD method and investigated the effect of the MWCNT length and distribution on the surface properties [11].

Singh et al. produced an effective electromagnetic wave absorber using MWCNTs coated onto CF surfaces by the electrophoretic deposition method [14]. As an approach to achieve chemical bonding between a MWCNT coating and CFs, Zhao et al. used melamine to realize chemical bonding between MWCNTs and CFs, and evaluated the interfacial properties using a microdroplet method [15]. Wu et al. prepared MWCNT-grafted CFs using a reactive, bi-functional, low-molecular-weight chemical, 3-aminopropyltriethoxysilane (APS) [16]. These two approaches utilize the chemical reaction between carboxylic acid on the surfaces of MWCNTs and CF, and amine with a catalyst. The use of amines to achieve chemical bonding between MWCNTs and CFs is of interest; however, it should be noted that the amount of MWCNTs on CFs is small, and not sufficient for scanning electron microscopy (SEM) observation [15,16]. Gamage et al. reported the enhancement of an electromagnetic wave absorber with CF fabric by MWCNT coating [18]. Despite many former challenges, there have been difficulties with the complex process of the CVD of MWCNTs on CFs and the electrodeposition of MWCNTs on CFs. In addition, there has been no strong chemical bonding reported, especially with methods such as the spray coating or dip-coating of MWCNTs onto CF. Table 1 shows the surface modifications to the CF surface.

There is a functional group that has high reactivity and an irreversible reaction with carboxylic acid and without the need for a catalyst, which is the oxazoline group, and there are low- and high-molecular-weight chemicals that include oxazoline groups. With regard to reactive polymers that include oxazoline, a fundamental polymerization study using 2-substituted-2-oxazoline was reported [21]. There are two typical reactive polymers that include oxazoline, poly-2-vinyl-2-oxazoline [22] from the polymerization of the 2-vinyl-2-oxazoline monomer, and poly-2-isopropenyl-2-oxazoline [23,24] from the polymerization of the 2-isopropenyl-2-oxazoline (Pipozo) monomer. There is a commercially available copolymer of 2-isopropenyl-2-oxazoline that has been utilized as a water soluble cross-linking agent [25]. However, reactive polymers including oxazoline have not typically been utilized for surface treatment and surface modification to date.

We conducted research on the surface modification of carbon materials using reactive polymers including oxazoline, which has high reactivity and an irreversible reaction with carboxylic acid present on carbon materials, with the aim to enhance the mechanical properties of composites. We previously conducted a quantitative investigation of the uniform formation of a monolayer reactive polymer, and its reacted and unreacted oxazoline on diamond particle surfaces using poly-2-vinyl-2-oxazoline [22]. We also found that a uniform coating of MWCNTs on the diamond particle surface through uniform formation of a monolayer reactive polymer of poly-2-vinyl-2-oxazoline with carboxylic acid on the MWCNT surfaces and unreacted oxazoline. However, there have been no reports about the effective modification of CFs with a large amount of MWCNTs through chemical bonding and its effect on the mechanical properties of the composite, which is melt-mixed based on the thermoplastics.

In the present study on the effective modification of CFs with a large amount of MWCNTs, i.e., physical anchoring, we attempted a first layer fabrication of a copolymer of Pipozo on CF (after elimination of sizing agents with an organic solvent) and a second layer fabrication of a uniform MWCNT coating on the first layer. To realize stronger bonding between MWCNTs and CFs through Pipozo, we focused on using not only carboxylic acid on MWCNTs and CFs, but also phenolic OH on MWCNTs and CFs to achieve more reaction sites with oxazoline, which could result in the formation of amide ester and amide ether groups, respectively. We conducted a quantitative analysis of the MWCNTs on CFs using UV-visible spectroscopy and SEM observation. The effect of the interface structure on the mechanical properties of the composite was investigated by melt-mixing of CFs and a polystyrene (PS) matrix. PS was used as a non-reactive matrix with high solubility in organic solvents for the surface analysis of CFs by elimination of the matrix after melt-mixing. PS composites with MWCNTs chemically bonded to CFs were prepared by melt-mixing and the tensile strength and tensile modulus were evaluated to identify the interfacial effect of MWCNTs bonded on the CF structure.

## 2. Materials and Methods

### 2.1. Materials

CF (T-700-12K, Toray Industries, Inc., Tokyo, Japan) was used after washing with acetone and ethanol to eliminate sizing agents. MWCNTs (NC-7000, Nanocyl SA, Sambreville, Belgium) [26] were used after the milling treatment with beads (RMB, Aimex Co., Ltd., Tokyo, Japan; zirconia beads, diameter = 0.5 mm, 1000 rpm) for 1.5 h to break up agglomerates and achieve dispersion of individual MWCNTs in N-methyl-2-pyrrolidone (NMP; Kanto Chemical Co., Inc., Tokyo, Japan) solvent. A copolymer of 2-isopropenyl-2-oxazoline (EPOCROS^®^ WS-300 10 wt% in water, Pipozo, 86 mol% oxazoline units in a copolymer Nippon Shokubai Co., Ltd., Osaka, Japan) [25] was used as the reactive polymer that includes oxazoline functional groups. PS (G100C, Toyo Styrene Co., Ltd., Tokyo, Japan) was used as a matrix for the composites because it is unreactive with MWCNTs and CFs, and is soluble in organic solvents, which facilitates elimination of the matrix [27].

### 2.2. Model Reaction of Phenol-OH and Oxazoline Using Low-Molecular-Weight Compound

Although CFs and MWCNTs are inert carbon materials, the surface of commercially available CFs is intentionally oxidized to enhance the adhesive interface and that of MWCNT also includes oxygen by the presence of oxygen impurities [7,9,15,16,28,29,30,31,32]. The most typical reaction-available acid functional groups by oxidation are COOH and phenol OH. There have been reports on the presence of acid functional groups such as COOH and phenol OH, and quantitative analyses have also been frequently reported [33,34,35,36]. The utilization of these acid functional groups for bonding and reaction has also been previously reported [9,15,16]. In this study, we focused on the use of the oxazoline reactive functional group, which has highly irreversible reactivity with COOH and phenol OH.

Evidence of the reaction between COOH or phenol OH, from the surface of CFs or MWCNTs, and oxazoline should be obtained using attenuated total reflectance-Fourier transform infrared (ATR-FTIR) spectroscopy. However, because of the absorption by carbon sp^2^ hybrid orbitals and the limited surface area, evidence of the bonding could not be obtained. Therefore, we used diamond particles with carbon sp^3^ hybrid orbitals having COOH and phenol OH acid functional groups as a model carbon material instead of CF and MWCNT. When the particle size was less than 1 μm, evidence that bonding was successfully detected using FTIR to confirm the reaction between oxazoline and COOH [22]. Therefore, based on the evidence that there are COOH and phenol OH on the surface of CFs and MWCNTs, and the evidence of FTIR using the diamond particles, we consider that this indicates that oxazoline reacts with COOH or phenol OH.

In the present experiments, we attempted to achieve a chemical reaction between oxazoline (from WS300, Nippon Shokubai Co., Ltd., Osaka, Japan) and phenol-OH, in addition to the reaction between oxazoline and carboxylic acid, which has already been confirmed to be 100% reaction at 100 °C after 3 h using low-molecular-weight oxazoline and nonanoic acid [22]. To examine the chemical reaction between oxazoline and phenol-OH in the present study, phenyl oxazoline (Tokyo Chemical Industry Co., Ltd., Tokyo, Japan) and 3-methoxy phenol (Tokyo Chemical Industry Co., Ltd., Tokyo, Japan) were used as low-molecular-weight model chemicals. The reactivity was evaluated using ^1^H nuclear magnetic resonance spectroscopy (^1^H-NMR; JNM-EC500, JEOL, Tokyo, Japan, 500 MHz) after 0, 1, 3, 6, and 24 h at 200 °C. Table 2 summarizes the model reaction compounds.

### 2.3. Analysis of Acid Groups on CF by the Neutralization Titration Method

Neutralization titration using sodium hydroxide and sodium hydrogen carbonate can provide a quantitatively accurate number of the carboxylic acid and phenol-OH groups on MWCNTs [31,32,33]. We already established the accuracy of this method by comparison with previously reported results [22]. Firstly, 10 g of CFs was washed with acetone and ethanol, and then treated with 0.01 M NaOH solution (500 mL) to determine the total acid functional groups (COOH and phenol-OH). The CFs were removed by filtration. 400 mL of 0.01 M HCl aqueous solution was added to 400 mL of the NaOH aqueous solution after CF filtration. The neutralization titration was performed using 0.002 M NaOH aqueous solution. In the same way, sodium hydrogen carbonate was used instead of sodium hydroxide to determine the COOH functional groups. The titration was carefully performed using a blank standard water sample to eliminate the effect of carbon dioxide dissolved in the water.

### 2.4. Layer-By-Layer Grafting of MWCNTs onto CFs

Uniform dense coating of MWCNTs on CFs with chemical bonding using Pipozo was conducted using a layer-by-layer method to obtain 2 layers. The first layer was a layer of the reactive polymer, Pipozo, on CFs through chemical bonding using a Pipozo aqueous solution at 80 °C for 1 h. This process gave the chemical reaction between the oxazoline of Pipozo and COOH present on the CF surfaces. The unreacted Pipozo was then completely removed by washing with methanol, followed by drying for 30 min at 100 °C. This provided a uniform monolayer layer of the reactive Pipozo, including unreacted oxazoline groups, which is described as CF/Pipozo. Due to the small surface area of CFs, quantitative evaluation of Pipozo using thermogravimetric analysis (TGA; TG-DTA8122, Rigaku Cooperation, Tokyo, Japan) is not possible.

A MWCNT dispersion was then prepared using 1-methoxy-2-propanol (PGME; Kanto Chemical Co., Inc., Tokyo, Japan) as a solvent with polyvinylpyrrolidone (PVP; Mw = 40,000, Tokyo Chemical Industry Co., Ltd., Tokyo, Japan) as a dispersant with the composition PGME 99.4 wt%, PVP 0.4 wt%, and MWCNT 0.2 wt%. CF/Pipozo was added to the dispersion and treated at 100 °C for 1 h to allow for the chemical reaction between oxazoline (from unreacted oxazoline of the Pipozo layer on CFs) and COOH (from the MWCNT surfaces). Unreacted MWCNTs were completely removed by washing with methanol and then drying at 100 °C for 30 min, which resulted in the formation of the second layer of uniform dense MWCNTs.

**Figure 1:**
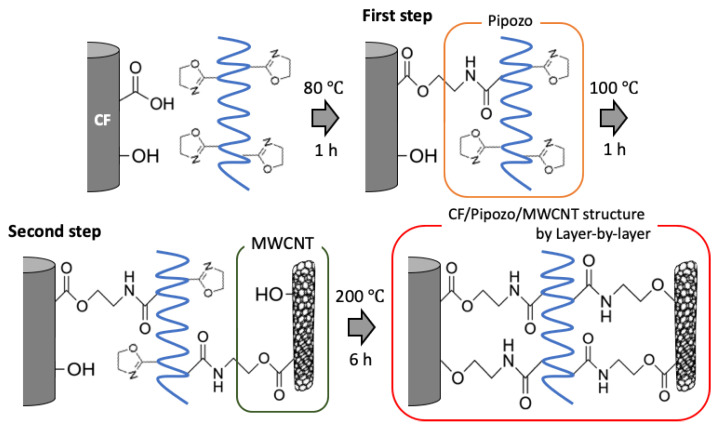
Schematic illustration of layer-by-layer coating Pipozo and MWCNTs onto CF surfaces by chemical reaction.

Under the present conditions, the chemical bonding for the two interfaces between CF and Pipozo, and between Pipozo and MWCNT, was only between COOH and oxazoline, i.e., without chemical reaction between phenol-OH and oxazoline, which requires a higher temperature for the reaction. To realize stronger bonding between MWCNTs and CFs through Pipozo, additional heat treatment at 200 °C for 6 h was performed to promote the reaction between phenol OH and oxazoline, between CFs and Pipozo, and between MWCNT and Pipozo. The MWCNTs were chemically grafted onto CFs through Pipozo, which is represented as CF/Pipozo/MWCNT, as illustrated in Figure 1.

### 2.5. Observation of CF/Pipozo/MWCNT Surface and Quantitative Evaluation of MWCNTs

**Figure 2:**
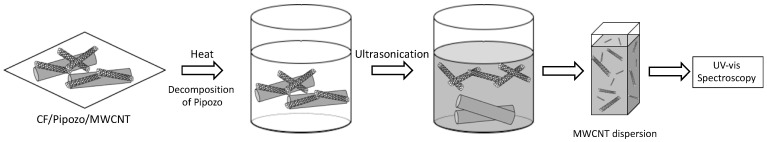
Process to remove MWCNTs (the second layer) from CF surfaces by Pipozo (the first layer) through the decomposition of Pipozo and the complete elimination of MWCNTs from the surfaces of the CFs by ultrasonication in a solvent for quantitative analysis of the amount of MWCNTs using UV-vis spectroscopy.

**Figure 3:**
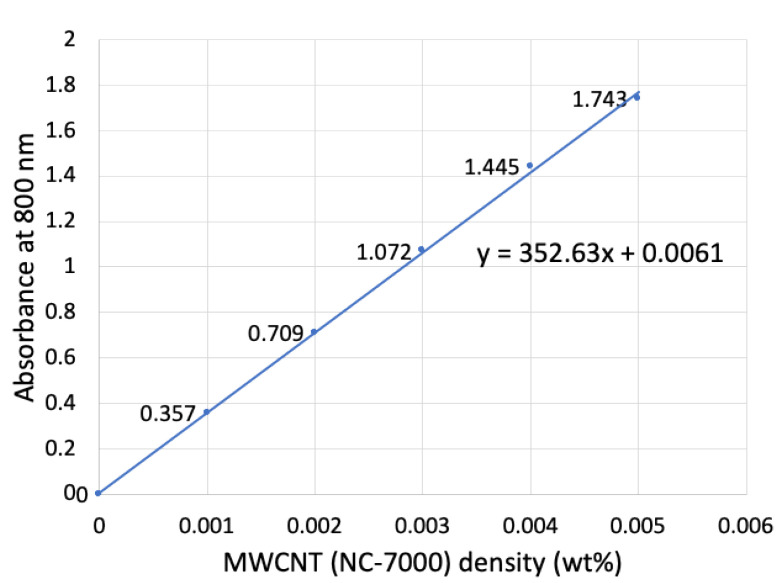
Calibration curve for MWCNT dispersions in NMP solvent.

SEM (JSM-7401F, JEOL Tokyo, Japan) was used for observation of the uniformly dense MWCNT-coated CFs. Quantitative evaluation of the amount of MWCNTs on the CFs was conducted using UV-vis spectroscopy measurements at 800 nm (U-4100, Hitachi High-Tech Corporation, Tokyo, Japan) of the MWCNT dispersion after removal of the MWCNTs from CFs by the decomposition of Pipozo and using a calibration curve of absorption with known MWCNT concentrations, as shown in Figure 2. Figure 3 shows the calibration curve for MWCNT concentrations of 0.001, 0.002, 0.003, 0.004, and 0.005 wt% with absorptions of 0.357, 0.709, 1.072, 1.445, and 1.743, respectively (calibration line equation: y = 352.63x + 0.0061).

The MWCNT dispersion for measurement was prepared from the MWCNTs eliminated from MWCNT-grafted CFs by the decomposition of Pipozo at 400 °C for 10 min using CF/Pipozo/MWCNT prepared by the layer-by-layer method. After the heat treatment, ultrasonic treatment (2 h) was conducted for complete removal of the MWCNTs from CFs and preparation of a stable dispersion in NMP solvent with PVP as a dispersant. The UV-vis absorption spectrum was measured twice using the prepared dispersion.

### 2.6. Fabrication and Physical Properties of PS Composites

Table 3 summarizes the three PS/CF composite samples prepared to evaluate the interfacial physical anchoring effect of the MWCNT-grafted structure, PS, PS-CF, and PS-CF/Pipozo/MWCNT. The PS composites were produced with the CF content (Pipozo layer/MWCNT layer) at 10 wt% using a batch type melt-mixer (Labo Plastomill Micro, Toyo Seiki Co., Ltd., Hyogo, Japan) at 200 °C and 30 rpm for 1 min. The mixed samples were crushed into small particles for hot compression molding to prepare mini dumbbell test pieces (45 mm × 5 mm × 0.5 mm) by pre-heat treatment at 200 °C for 15 min followed by treatment at 200 °C under 5 MPa pressure for 3 min, and then rapidly cooled at 5 MPa for 3 min using cold compression molding (Mini test press, Toyo Seiki Co., Ltd., Hyogo, Japan).

**Figure 4:**
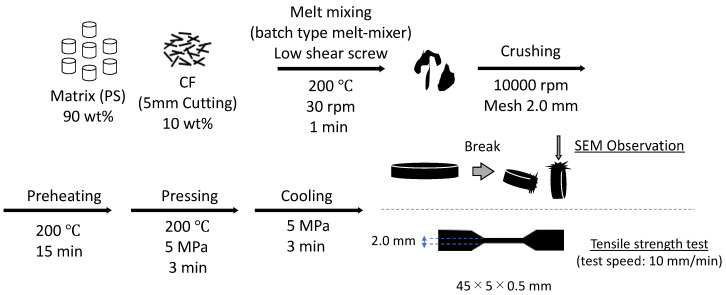
Process flowchart from the preparation of PS composites to SEM observation and evaluation.

Tensile tests were conducted using a vertical tensile test machine (MCT-1150, A&D Company Limited, Tokyo, Japan) with a crosshead speed of 10 mm/min (distance between chucks: 12 mm, width: 2 mm). The actual thickness was carefully determined from the average of three measurements. In addition, the fractured cross sections after freeze fracture were observed using SEM to evaluate the adhesion of the matrix resin onto the CF surface between the PS-CF and PS-Pipozo/MWCNT/CF composites. Figure 4 shows a process flowchart from the fabrication of the PS/CF composite to the evaluation of interface adhesion (SEM) and tensile testing.

## 3. Results and Discussion

MWCNT was strongly bonded onto CF with an aim to improve the mechanical properties of the composite through a physical anchoring effect, which requires strong bonding between MWCNTs and CFs with the first layer of the reactive Pipozo polymer. It is essential to confirm the reactivity between oxazoline and COOH, together with that between oxazoline and phenol-OH. The chemical reaction between Pipozo and CFs, related with the first layer, is discussed based on the model reaction, together with a quantitative analysis of the acidic functional groups on the CF surface (3-1). The amount of MWCNTs, which is the second layer and is chemically bonded to the first layer, is discussed based on the quantitative analysis (3-2). The mechanical properties of the composite using CF/Pipozo/MWCNT are also discussed with respect to the physical anchoring effect, together with the amount of MWCNTs remaining on the CF surface (3-3). It is important to confirm whether the presence of MWCNTs is maintained or not after melt-mixing of the composite to carefully interpret the effect on the mechanical properties; therefore, the SEM analysis of the surface after melt-mixing is discussed (3-4). The cross section of the composite using CF/Pipozo/MWCNT after freeze fracturing is also discussed with respect to the strong adhesion of the physical anchoring effect (3-5), and the effect of the interfacial properties on the tensile modulus in the case of short fibers is discussed (3-6). The results are thus described and discussed in depth with a focus on the interface formed by the simple layer-by-layer method.

### 3.1. Model Reaction Using Low-Molecular-Weight Compounds and the Surface Acid Groups of CFs and MWCNTs

**Figure 5:**
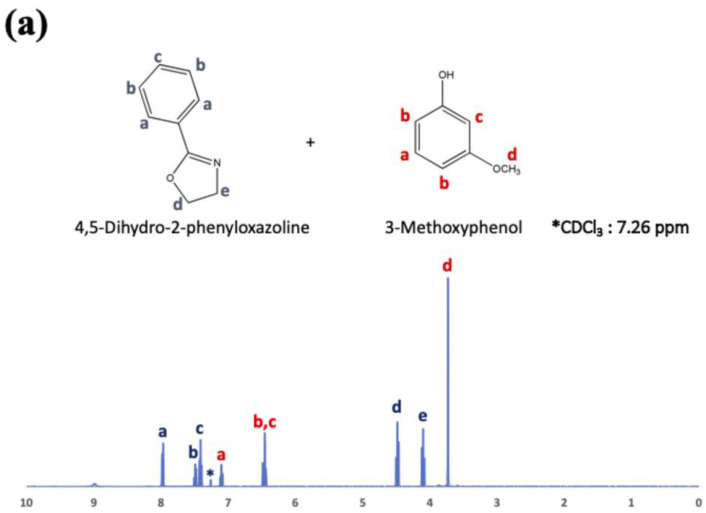

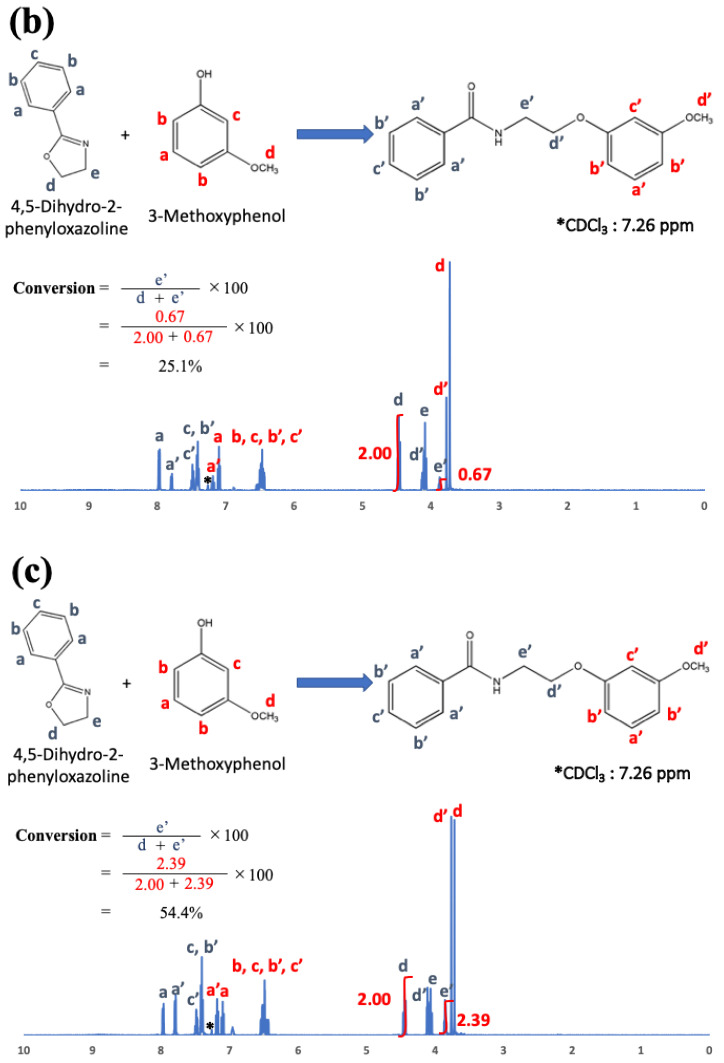

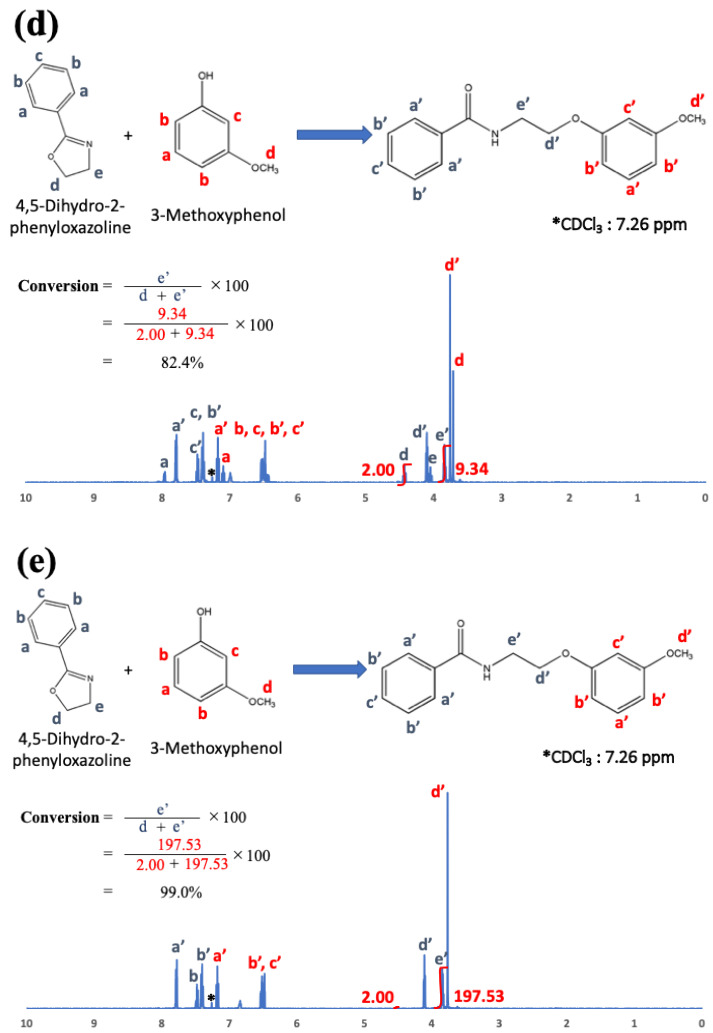

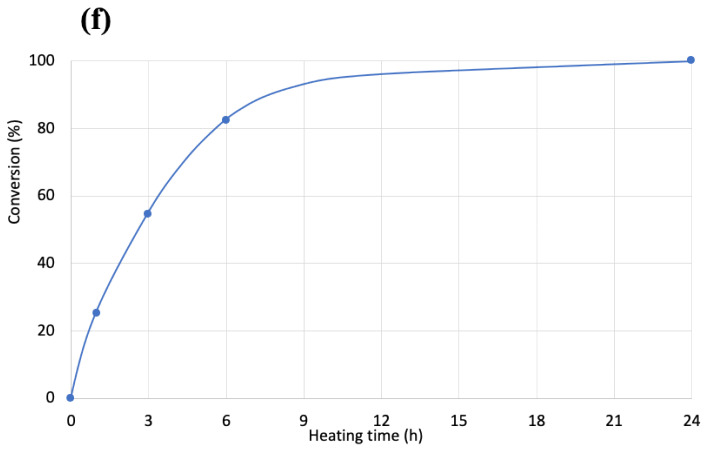
(**a**) ^1^H-NMR (500 MHz) spectra for the mixture of 4,5-dihydro-2-phenyloxazoline and 3-methoxyphenol immediately after mixing at room temperature, (**b**) after 1 h at 200 °C, (**c**) after 3 h at 200 °C, (**d**) after 6 h at 200 °C, and (**e**) after 24 h at 200 °C. (**f**) Reaction conversion based on ^1^H-NMR (500 MHz) measurements in (**a**–**e**) calculated from the integral areas of the d and e’ peaks.

COOH and phenol OH are present on the CF surface, both of which can react with Pipozo, which results in the formation of the first layer produced using the layer-by-layer method. As a fundamental experiment, it is important to investigate the reaction conditions, such as the temperature and time, and the resultant reaction rate between COOH and oxazoline, and between phenol OH and oxazoline (summarized in Table 1). We already performed a model reaction between COOH and oxazoline using 2-ethyl-2-oxazoline and nonanoic acid, which indicated that reaction at 100 °C for 3 h provided a complete reaction, i.e., the formation of an amide ester bond [22]. In the present study, the model reaction between phenol OH and oxazoline was conducted using 3-methoxy phenol and phenyl oxazoline as model low-molecular-weight compounds. The reaction rate was evaluated using ^1^H-NMR, and the results are shown in Figure 5a–e just after mixing (a), and after 1 h (b), 3 h (c), 6 h (d), and 24 h (e). Figure 5 indicates that phenol OH can react with oxazoline at 200 °C for 6 h to form amide ether (>80% of reaction rate). Therefore, the reaction conditions (100 °C for 3 h) for COOH and oxazoline do not allow the reaction between phenol OH and oxazoline.

It is important to evaluate the acidic functional groups, COOH and phenol OH, on the CF surface by neutralization titration. Let us discuss the accuracy and the reliability of the neutralization titration method by comparison with previous studies using similar commercially available MWCNTs [34,35,36]. The MWCNTs used in the present study showed a total acid (COOH and phenol OH) content of 7.4 × 10^−4^ mol/g. Ackermann and Krueger reported a total acid content of 3.0 × 10^−4^ mol/g [33] and Zhang et al. reported a total acid content of 2.0 × 10^−4^ mol/g [36]. Based on careful comparison, the total number of acid functional groups was evaluated to be almost identical, which suggests the neutralization titration method is sufficiently accurate. The same method was applied to evaluate the acidic functional groups on the CF surface.

From the neutralization titration method using sodium hydroxide and sodium hydrogen carbonate, the amounts of COOH and phenol OH were determined to be 0.45 × 10^−6^ mol/g and 1.13 × 10^−6^ mol/g, respectively. It is important to evaluate the density of acidic functional groups per unit area on the CF surface to confirm the excess amount of oxazoline groups compared with the total acid group content for utilization of the unreacted oxazoline with the MWCNTs. From the 7 μm diameter of the CFs and the density of 1.8 g/cm^3^, the unit area was set to be 1 × 1 nm because the C-C bonding distance is 0.15 nm and the calculation was already performed for the diamond particle surface in our previous work [22]. It was calculated that the number of COOH groups per unit area is ca. 0.8, and that of phenol OH is ca. 2.1, which are similar to that on a diamond particle surface [22]. The number of oxazoline molecules per unit area after monolayer reaction formation on a diamond surface was ca. 10 and that on the CF surface would most probably be similar, which suggests that the number of oxazoline molecules is more than that of the total number of acid groups and that the unreacted available oxazoline should remain after formation of the first layer.

Table 4 summarizes our experimental evidence of the acid groups by the neutralization titration and the results from the references [33,36,37,38,39] about the acid groups by the neutralization titration and the oxygen atomic percent by XPS measurements, in which the untreated MWCNTs are used for all results. Regarding the experimental evidence of the presence of the acid functional groups on the untreated MWCNT, we carried out the quantitative analysis of the total acid groups (COOH and phenol OH) according to the established former method using neutralization titration [33,36]. The experimental evidence showed that even the untreated MWCNT has the acid functional groups with 7.4 × 10^−4^ mol/g. This value is almost in good agreement with the acid functional groups (1.0 × 10^−4^ mol/g, 2.0 × 10^−4^ mol/g) from the untreated MWCNT in the former articles [33,36]. In addition, based on the former results from the references [37,38,39], the evidence of the presence of oxygen on the surface was clearly shown as the atomic percentage (1.4–1.88) even for the commercially available untreated MWCNTs. From our result and the former references, the presence of the acid and the oxygen of the commercially available untreated MWCNTs was demonstrated.

### 3.2. Quantitative Evaluation of MWCNT Amount by the Layer-By-Layer Method

**Figure 6:**
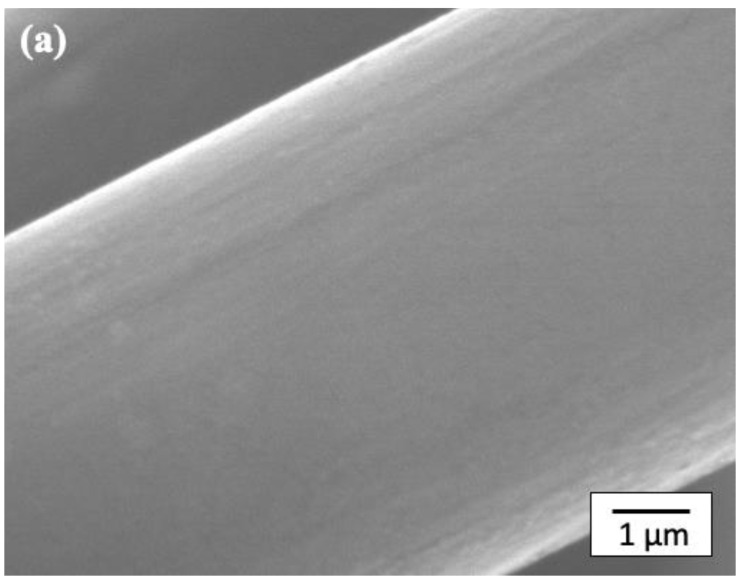

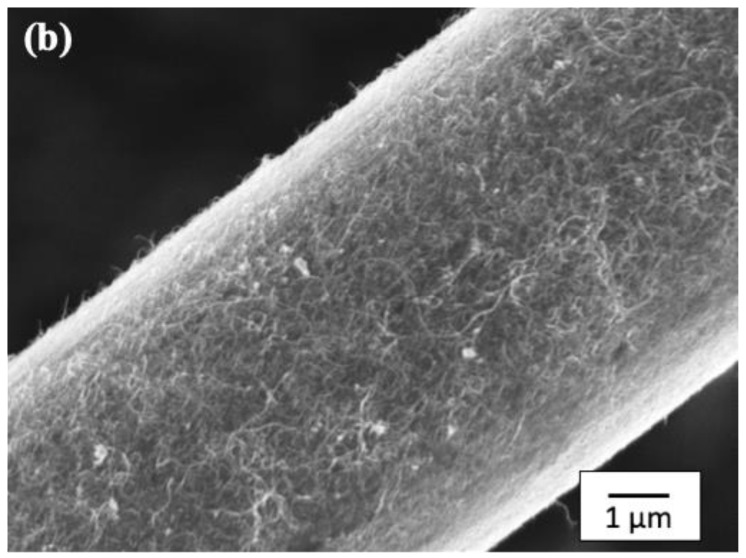
SEM images of the CF surface using the layer-by-layer method. (**a**) CF surface coated with Pipozo (after the first layer, the Pipozo layer was too thin and smooth to be visible by SEM). (**b**) CF surface coated with MWCNTs (after the second layer, and solvent washing several times).

Figure 6 shows SEM micrographs of the CF surface after the layer-by-layer method. Figure 6a shows an SEM micrograph of a CF with the first layer of Pipozo, i.e., CF/Pipozo, which suggests a very smooth surface that originates from the CF surface. This indicates that the first layer of Pipozo is a uniform monolayer structure due to the reactivity of Pipozo, i.e., oxazoline and COOH on the CF surface and the complete washing process of unreacted Pipozo after the first layer reaction. TGA could not detect degradation of the first layer using CF/Pipozo, at least within the detection limit. In our previous study [22] using diamond particles, the uniform monolayer structure was formed by poly-2-vinyl-2-oxazoline, which is similar to Pipozo. TGA analysis could not detect the weight loss of poly-2-vinyl-2-oxazoline due to the limitation of the equipment when using 40 μm-diameter diamond particles, because the surface area is very small. However, when using 1 μm-diameter diamond particles, the weight loss due to the degradation of poly-2-vinyl-2-oxazoline was detectable, which suggests the thickness of the layer was ca. 1–2 nm. The uniformity was demonstrated using MWCNTs as a marker [22]. Therefore, it was considered that a similar uniform monolayer of Pipozo was formed on the CF surface.

Figure 6b shows an SEM micrograph of MWCNTs, the second layer, chemically bonded with Pipozo, the first layer, chemically bonded with the CF surface. This SEM observation was conducted after a complete washing process to remove unreacted MWCNTs, and suggests that there is a uniform monolayer of Pipozo on the CF surface, which resulted in a very dense and large amount of MWCNTs that formed the uniform coating of the second layer. It should be noted that there are several previous reports [15,16] on MWCNT-coated CFs based on SEM observations; however, all of these reports showed only a small amount of MWCNTs that was much less than observed in the present work. No research to date has shown such a uniform and large amount of MWCNTs chemically bonded on the surface of CFs.

Next, quantitative analysis of the amount of MWCNTs on CFs indicated a substantially large amount with uniformity. For this evaluation, a calibration curve for UV-vis absorption at 800 nm as a function of known MWCNT dispersion concentration was produced. Figure 2 shows the preparation method and how the MWCNTs were removed from the CFs to make a dispersion of MWCNTs. The absorption was measured to be A = 0.205, so that the concentration was determined to be x = 5.60 × 10^−4^ wt% from the calibration curve.

Here, the solution of PVP dissolved in NMP had the composition (NMP: 9.817 g, PVP: 0.2014 g, Total: 10.0184 g), so that the amount of MWCNTs was calculated to be 5.61 × 10^−5^ g. The CF/MWCNT amount was 0.0113 g and that of the MWCNTs was 5.61 × 10^−5^ g; therefore, the MWCNT weight percentage based on the CF/MWCNT (CF plus MWCNT is 100%) was calculated to be 0.50 wt%. This was measured twice in the same way to obtain an average. The amount of MWCNT coating based on the CF was thus evaluated to be 0.44 ± 0.06 wt%.

To achieve a deeper insight into the quantitative reaction by the layer-by-layer method, it is important to determine the mole number of COOH, phenol OH, oxazoline (reacted), and oxazoline (unreacted) in the first layer because the substantial amount of unreacted oxazoline remaining on the first layer is critical to react with the COOH and phenol OH groups of the MWCNT surfaces for formation of the second layer. Our previous study with diamond particles suggested that the total reacted (with COOH and phenol OH) and unreacted oxazoline were 9.1, 2.3, and 6.8, per unit area (1 × 1 nm), respectively [19,22].

With the various similarities of acidic functional groups (1–2 units) per unit area (1 × 1 nm), the preparation process and the resultant uniform monolayer between diamond particles and CFs, a noticeable amount of unreacted oxazoline remained in the first uniform layer of Pipozo. This could lead to a substantial amount of MWCNTs on the CFs (0.44 wt%), where the CF surface is not visible due to the coated MWCNTs, which is quite in contrast with the small amount of MWCNTs coated on CFs, where the CF surface was visible in the previous studies [15,16]. Therefore, the first reactive polymer layer has very high reactivity and is quite uniform. The large amount of MWCNTs is considered to be due to physical anchoring of the interfacial effect in the composite, which improves the mechanical properties.

### 3.3. Mechanical Properties of PS Composites

**Figure 7:**
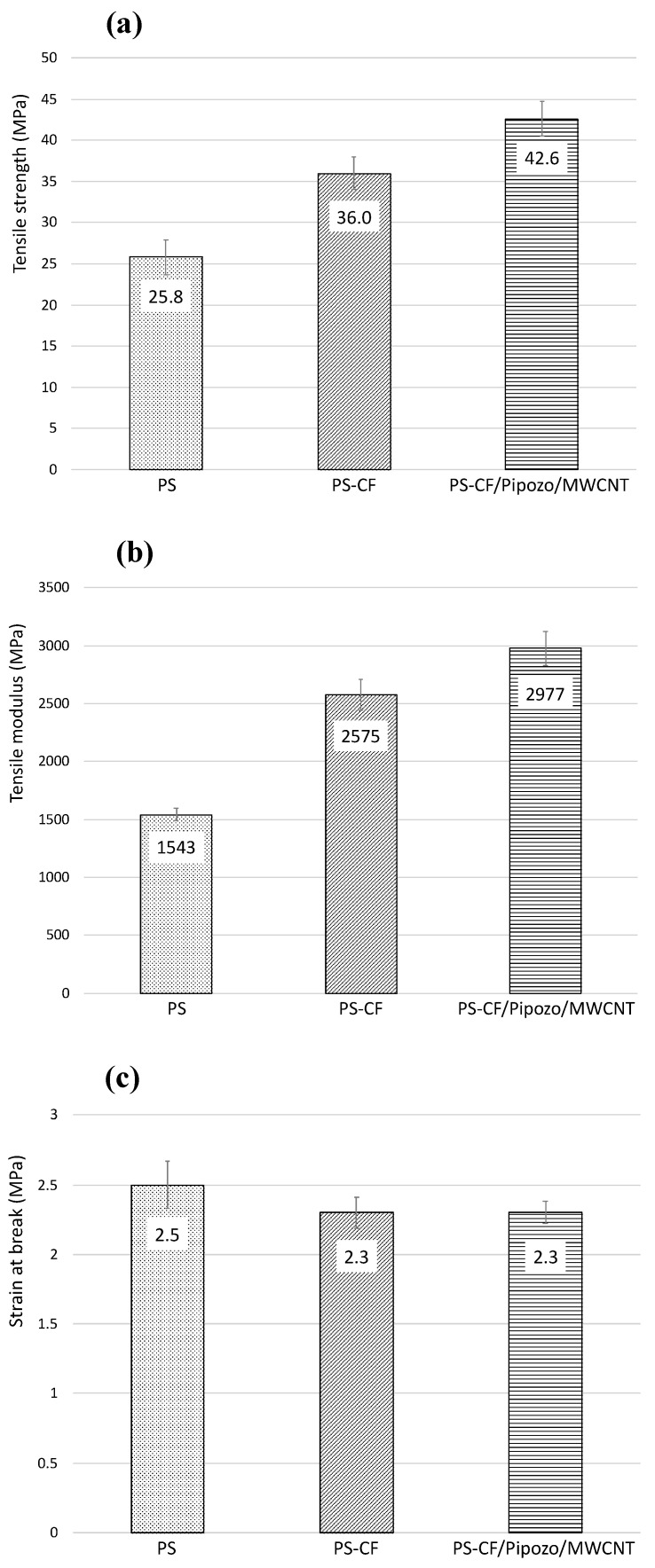
Mechanical properties of various PS composites (PS, PS-CF, PS-CF/Pipozo/MWCNT); (**a**) tensile strength, (**b**) tensile modulus, and (**c**) strain at break.

Figure 7 shows the tensile strength test results for the composites, i.e., tensile strength, tensile modulus, and elongation at break. From Figure 7a, the addition of 10 wt% CF into PS resulted in a 40% increase of the strength and a 70% increase of the modulus. This increase is due to the reinforcement effect of CFs. It is suggested that the increase in the mechanical properties by the addition of CFs is much larger in PS than in polypropylene, which originates from the interface, i.e., the interaction of π–π stacking. There is a substantial amount of MWCNTs (0.4 wt%) at the interface of PS-CF/Pipozo/MWCNT, which leads to large surface unevenness and the physical anchoring effect. The MWCNT layer on the CF surface enhanced the tensile strength with a 20% improvement and the tensile modules with a 20% increase. Figure 7c shows an almost similar elongation at break. The improvement of the tensile strength and modulus is considered to be due to the surface unevenness, i.e., the physical anchoring effect, together with the interaction of π–π stacking.

Careful analysis of the amount of MWCNTs indicated 0.44 wt% MWCNTs on the CFs (therefore, 0.044 wt% MWCNTs based on the total composite). It is notable that only 0.044 wt% MWCNT located at the CF surfaces resulted in such a significant improvement. It is unclear whether the presence of MWCNTs on CFs can be maintained or not after severe melt-mixing, which was the process used for preparation of the present PS composite.

### 3.4. SEM Observation of MWCNT-Coated CF Surface after Melt-Mixing

**Figure 8:**
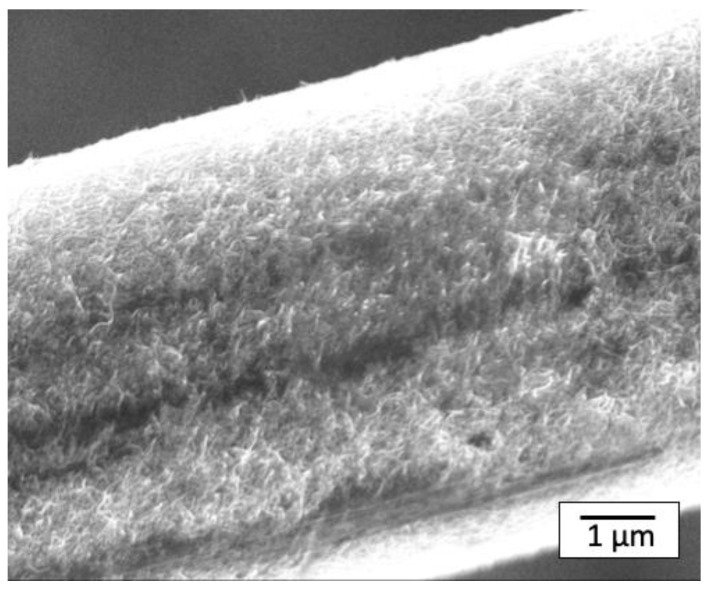
SEM image of the MWCNTs remaining on the CF surface. The sample was prepared using CF/Pipozo/MWCNT (layer-by-layer method, Figure 5), which was melt-mixed with PS and PS was then completely removed with a solvent treatment several times. This demonstrates that the MWCNTs (second layer) were strongly bonded to CF by Pipozo (first layer) and the MWCNTs were not removed by the melt-mixing process.

There have been several reports on MWCNT-coated CF composites, which are mostly based on thermosetting resin composites, where liquid epoxy penetrates into the CF surface. In the penetration process, the force near the CF surface is limited because the thermosetting resin is a relatively low viscosity liquid. On the other hand, there are a few previous studies on thermoplastic matrix composites [12,13,19]. As an example, Rahmanian et al. produced MWCNTs grown by the CVD method from the CF surface, and composites were prepared using polypropylene [40]. In this case, the CF received a large shear force due to the high viscosity of the molten resin so that MWCNTs could be eliminated from the CFs during melt-mixing. However, there was no investigation of the CF surface by observation after melt-mixing. It is thus necessary to confirm the presence of MWCNTs on CFs after melt-mixing, which can be simply checked by elimination of the PS matrix with the use of an organic solvent, such as dichloromethane (Kanto Chemical Co., Inc., Tokyo, Japan). Elimination of the matrix was performed and the surface of the CF was observed using SEM. Figure 8 shows an SEM image of the CF surface after washing the composite (PS-CF/Pipozo/MWCNT), which revealed that a substantial amount of MWCNTs still remained even after melt-mixing. An additional question arises as to whether the matrix PS resin really penetrates into the MWCNT structure. To answer this question, SEM observations of cross sections after freeze fracturing were conducted.

### 3.5. SEM Observation of Fracture Cross Sections of the Composite

The improvement of the mechanical properties of the composites can be correlated with the fracture patterns around the surface of CFs. The fracture of the composite with weaker mechanical properties was initiated at the interface, i.e., the weak point, which resulted in a smooth CF surface. On the other hand, that with stronger mechanical properties was initiated in the matrix because of the strongly adhesive interface. There have been no reports on the effect of physical anchoring on the surface of CFs on the fracture phenomenon.

**Figure 9:**
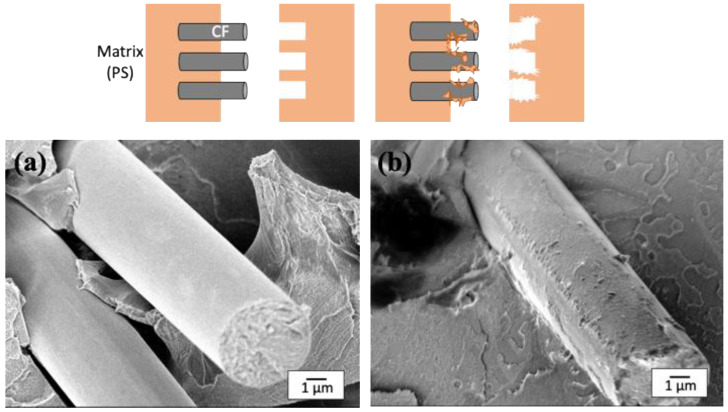
SEM images of freeze-fractured surfaces of the composites after impact tests; (**a**) PS-CF composite (interfacial peeling) and (**b**) PS-CF/Pipozo/MWCNT composite (cohesive failure).

The effect of the uneven structure by the substantial amount of MWCNTs resulted in a strong chemical bonding to the CF surface, as evidenced by the fracture cross section near the CF; Figure 9 shows SEM images of the fracture cross sections of two composites (PS-CF, PS-CF/Pipozo/MWCNT), together with the two types of model structure, interfacial peeling and cohesive failure. The fracture surface of the CF in the PS-CF composite was smooth, which suggests the failure occurred at the interface. On the other hand, for the PS-CF/Pipozo/MWCNT composite, there was PS resin remaining on the surface, which suggested the failure occurred in the matrix, i.e., cohesive failure. This suggests that PS resin penetrated into the MWCNT layer, which resulted in strong adhesion at the interface.

### 3.6. Effect of Interface on Tensile Properties

The properties of the interface between the matrix and CF are often reported with respect to the microdroplet or interfacial shear strength measurements using long fibers [7,9,11,15,16]. However, these are for long fibers, and the interfacial properties have not been evaluated using short fibers. The strength of the interface between the matrix and CF can be evaluated from the length of the CF and the tensile modulus of the composite [41]. The fiber length in the composite was measured to show that there is no difference in fiber length. For the CF length measurements, approximately 300 fibers were randomly measured and their number and weight averages were calculated. For PS-CF, the number average fiber length was 357.6 ± 142.0 μm and the weight average fiber length was 385.1 μm. For PS-CF/Pipozo/MWCNT, the number average fiber length was 329.3 ± 138.6 μm and the weight average fiber length was 387.4. There was no significant difference in CF length between these two samples. From the theoretical equation, it can be shown that the difference in CF length was due to the effect of the interface of the MWCNTs coated on the CF because there was no difference in CF length, i.e., the increase in the mechanical strength of the composite was due to the action of the MWCNTs at the interface.

The current improvement of the mechanical properties was approximately 20% and further improvement is expected in future work by optimization of the amount of MWCNTs and the structure (e.g., diameter, length, and linearity). These results for the effective modification of CFs by MWCNTs with a simple process to enhance the mechanical properties may be applicable for all types of thermoplastic polymers, especially engineering plastics that will meet the requirements of the automotive industry.

## 4. Conclusions

MWCNTs were grafted onto the surface of CFs using a reactive polymer including oxazoline. The process, called layer-by-layer, consists of two steps, i.e., coating with the reactive polymer and the MWCNT layer process. A substantial number of MWCNTs were grafted onto CFs by this simple process, which almost completely remained after melt-mixing with PS as a thermoplastic, as confirmed after the elimination of PS with a solvent. This is the first evidential observation of the interfacial structure. The effective surface modification led to an improvement of the mechanical properties, which was supported by SEM observation that showed that fracture was initiated in the matrix instead of the interface. Further improvement will be expected by optimization of the amount of MWCNTs and the structure. This interfacial concept may be applicable to short CF composites based on various thermoplastics, which should meet the requirements for lightweight applications in the automotive industry.

## Figures and Tables

**Table 1:** Surface modification using MWCNTs on CFs.

	Without MWCNTs	With MWCNTs			
Method	Chemical bonding	Chemical vapor deposition (CVD)	Electrophoretic deposition (EPD)	Chemical bond	Dip coating
Reference	[7,8,9]	[10,11,12,13]	[14]	[15,16]	[18,19]

**Table 2:** Model reaction of oxazoline and acidic functional groups (COOH and phenol OH groups) and its application to surface modification, i.e., reaction between poly(2-isopropenyl-2-oxazoline) and COOH and phenol OH groups on the CF surface.

	Oxazoline	Acid	Evaluation of Chemical Reaction
(1) Model reaction with low-molecular-weight compounds.	2-Ethyl-2-oxazoline 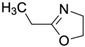	Nonanoic acid 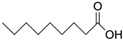	^1^H-NMR (evidence of quantitative reaction amount)
(2) Model reaction with low-molecular-weight compounds.	Phenyl-oxazoline 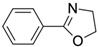	3-Methoxyphenol 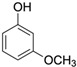	^1^H-NMR (evidence of quantitative reaction amount)
(3) High reactivity oxazoline and acid functionality onto CF.	Polyisopropenyloxazoline-copolymer(Pipozo) 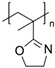	Acid functional groups (such as COOH) on the surface of CF, MWCNT interface	Layer-by-Layer 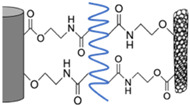

**Table 3:** Prepared samples and their abbreviations.

	Abbreviation	PS (wt%)	CF (wt%)	CF/Pipozo/MWCNT (wt%)
Samples	PS	100	0	0
	PS-CF	90	10	0
	PS-CF/Pipozo/MWCNT	90	0	10 (Pipozo *: n.d, MWCNT **: 0.04 wt%)

Remarks * Not detectable by TGA; ** Determined from Figure 3.

**Table 4:** Experimental evidence and the former results from the references [33,36,37,38,39] about the total acid groups and the oxygen atomic percentage from the neutralization titration and XPS, respectively, using the commercially available untreated MWCNTs.

	Neutralization Titration; Total Acid (-COOH, Phenol-OH) Functional Groups	XPS; Oxygen Atomic % Surface	Product Name of Commercial MWCNT
Experimental value of this study	7.4 × 10^−4^ mol/g	-	NC7000^TM^ (untreated) from Nanocyl SA
Reference [33]	1.0 × 10^−4^ mol/g	-	MWCNT(untreated) from FutureCarbon GmbH
Reference [36]	2.0 × 10^−4^ mol/g	-	MWCNT(untreated) from Cheap Tubes Inc.
Reference [37]	-	1.5 atomic%	NC7000^TM^ (untreated) from Nanocyl SA
Reference [38]	-	1.4 atomic%	NC7000^TM^ (untreated) from Nanocyl SA
Reference [39]	-	1.88 atomic%	NC7000^TM^ (untreated) from Nanocyl SA

## Data Availability

The raw data presented in this study are available on request from the corresponding author.
